# Self-rated familiarity with autism spectrum disorders among practicing nurses: a cross-sectional study in the palestinian nursing practice

**DOI:** 10.1186/s12912-021-00764-3

**Published:** 2021-12-03

**Authors:** Ramzi Shawahna

**Affiliations:** 1 Department of Physiology, Pharmacology and Toxicology, Faculty of Medicine and Health Sciences, An-Najah National University, New Campus, Building: 19, Office: 1340, P.O. Box 7, Nablus, Palestine; 2 An-Najah BioSciences Unit, Centre for Poisons Control, Chemical and Biological Analyses, An-Najah National University, Nablus, Palestine

**Keywords:** Autism spectrum disorder, Familiarity, Knowledge, Nurses, Palestine

## Abstract

**Background:**

Autism spectrum disorders (ASDs) are one of the most prevalent neurodevelopmental disabilities of early childhood. Practicing nurses are in a key position to help patients with ASDs and/or their caregivers/families. This study was conducted to assess self-rated familiarity with ASDs among practicing nurses in Palestine. The study also aimed to identify the sociodemographic and practice variables that could predict high self-rated familiarity scores.

**Methods:**

This was a cross-sectional study using a questionnaire. The study was conducted in the period between January 2019 and May 2019. The questionnaire collected: 1) the sociodemographic, pedagogic, and practice variables of the nurses, 2) their self-rated familiarity with signs and symptoms, treatment options, and community resources of ASDs, 3) their self-rated confidence in their abilities to provide counseling for parents/family/caregivers on the drugs prescribed for children/patients with ASDs and their potential adverse effects, and 4) their willingness to receive education/training on issues in ASDs.

**Results:**

The questionnaire was completed by 357 practicing nurses. The practicing nurses self-reported inadequate familiarity with symptoms, treatment, and community resources of ASDs. The mean familiarity score was 35.8% (SD: 18.9%). The nurses also expressed low confidence in their ability to provide counseling services to caregivers/families of children with ASDs. About 75% of the nurses agreed that they could benefit from taking a continuing educational/training program in the area of ASDs and about 82% of the nurses agreed that the nursing school curriculum should include courses in the area of ASDs. The multiple linear regression model showed that higher familiarity scores were predicted by having longer practical experience, having a higher academic degree in nursing, and having a continuing educational course/program on ASDs.

**Conclusion:**

Findings of this study highlighted inadequate familiarity with issues of ASDs among practicing nurses. Higher familiarity was predicted by the length of practical experience, higher academic degree in nursing, and having continuing educational course/program on ASDs. Specifically designed pedagogic interventions might be helpful in increasing familiarity of practicing nurses on ASDs. More investigations are still needed to evaluate if these interventions can improve familiarity and services provided to patients with ASDs.

**Supplementary Information:**

The online version contains supplementary material available at 10.1186/s12912-021-00764-3.

## Background

Autism spectrum disorders (ASDs) are one of the most prevalent developmental disabilities of early childhood. ASDs are characterized by atypical patterns of communication and social interactions in addition to observed restricted and repetitive behavior and interest [1]. Today, ASDs are believed to be the fastest growing neurodevelopmental disabilities in the world [2, 3]. According to recent statistics, about 1 child in every 68 has been diagnosed with a type of ASDs [4]. ASDs affect individuals from all ethnicities, racial, and socioeconomic groups [4]. Due to the increasing prevalence, ASDs are considered a public health concern in many healthcare systems around the world [5, 6]. Much of the efforts today are directed towards helping people with ASDs and their families.

Although ASDs were first reported in 1943, however, ASDs are still considered puzzling disorders because many issues in ASDs remain uncertain [7]. Therefore, it is highly likely that parents and caregivers of children with ASDs would seek advice from healthcare professionals. Healthcare professionals are expected to educate parents and caregivers on ASDs. Nurses are both respected and trusted healthcare professionals [8]. As the prevalence of ASDs is on the rise globally, nurses are expected to encounter many advice seeking parents of children with ASDs or caregivers of individuals with ASDs [9, 10]. Nurses are expected to help caregivers/families sort through information and resources to make better informed decisions concerning their child/patient with ASDs. To this end, nurses are expected to educate parents and caregivers on the different signs and symptoms of ASDs, drugs used to alleviate symptoms of ASDs, specific behavioral symptoms that medications seek to alleviate, the various side effects of these medications, and community resources allocated for ASDs in their regions that can be used for referral of a child/patient who is experiencing symptoms that can be linked to ASDs. Nurses should be in a key position to provide information and services to parents and/or families/caregivers on ASDs.

Palestinians receive healthcare services from three main sectors: healthcare facilities of the government, healthcare facilities of the private sector, and healthcare facilities of the United Nations Relief and Works Agency (UNRWA) for Palestine Refugees in the Near East. Nurses are main healthcare service providers in almost all primary, secondary, and tertiary healthcare facilities of the three sectors in Palestine. Nurses also provide services to individuals in nursing homes and home care. Additionally, nurses provide services in highly specialized healthcare facilities like those providing diagnostic, behavioral, cognitive, occupational, medication, and rehabilitation therapy/management services for patients with ASDs [6]. Typically, nurses are responsible for planning and providing medical and nursing care to patients with acute and/or chronic physical and/or mental illnesses.

Assessing familiarity of healthcare professionals on a certain health issue can serve as a quality measure in modern healthcare systems. A study conducted in the state of Virginia showed that school nurses were aware of issues relevant to ASDs, symptoms associated with ASDs, and medications used in the management of ASDs [11]. Regrettably, other studies have reported many cases of healthcare professionals lacking adequate familiarity with ASDs [9, 12–17]. A study among practicing pharmacists in Palestine reported low familiarity with ASDs [18]. More recently, knowledge gaps of issues in ASDs were also identified among Palestinian medical students [19]. It has been argued that healthcare professionals who lack adequate familiarity with ASDs are not expected to deliver optimal healthcare services and advice to families and caregivers of patients with ASDs. Because nurses are important healthcare providers in the Palestinian healthcare system, nurses are supposed to be adequately familiar with issues in ASDs in order to help caregivers/families of patients with ASDs.

To this date, practicing nurses in the Palestinian practice were not assessed for their familiarity with ASDs. Therefore, little is known on how practicing nurses in Palestine self-rate their familiarity with ASDs. Additionally, little on is known on how practicing nurses in Palestine self-rate their confidence in their abilities to provide counseling for parents/family/caregivers on the drugs prescribed for children/patients with ASDs and their potential adverse effects, willingness to receive education/training on issues in ASDs. The aims of this study were to: 1) assess self-rated familiarity of practicing nurses in Palestine with regard to ASDs, 2) assess self-rated confidence of practicing nurses in Palestine in their abilities to provide counseling for parents/family/caregivers on the drugs prescribed for children/patients with ASDs and their potential adverse effects, 3) assess willingness of practicing nurses in Palestine to receive education/training on issues in ASDs, and 4) identify the sociodemographic and practice variables that could predict high self-rated familiarity scores.

## Methods

### Design of the study

The current investigation was a cross-sectional study in the Palestinian nursing practice. The study was conducted in the period between January 2019 and May 2019. The present study adheres to the Strengthening the Reporting of Observational Studies in Epidemiology (STROBE) Statement for reporting cross-sectional observational studies as shown in Supplementary Table S1.

### Study nurses and sampling

The sample size needed for the present study was calculated using a calculator that is commonly used to compute sample sizes. The calculator can be accessed online through the link (www.raosoft.com). The calculator estimates sample sizes using Daniel’s formula [20]. For this study, the number of practicing nurses needed for this study was estimated for a population of 3000 nurses practicing in the different healthcare facilities across the West Bank of Palestine. The number of nurses was estimated at a 95% confidence interval (CI) and tolerating a default margin of error of 5%. For this study, 341 practicing nurses were needed. To ensure recruiting the sample size needed for this study, 400 nurses were invited to participate in this study. A total of 357 practicing nurses participated in this study, giving a high response rate of 89.3%.

Participants were registered nurses who were licensed to practice nursing by the Palestinian Ministry of Health. Practicing nurses were visited and recruited in their places of work. The principal investigator contacted practicing nurses through personal contacts in the main healthcare facilities across the West Bank of Palestine. These healthcare facilities were located in the different governorates of the West Bank: Jenin, Tulkarem, Tubas, Nablus, Qalqilya, Salfit, Ramallah and Al Bireh, Jericho, Jerusalem, Bethlehem, and Hebron. The principal investigator and the key contact nurses explained the objectives of the study to the potential participants and invited them to take part in the study. Nurses were included in this study when they met the following inclusion criteria: 1) were licensed to practice nursing in Palestine by the Ministry of Health, 2) were practicing in a healthcare facility that was visited by patients with ASDs either for acute and/or chronic physical and/or mental illnesses, 3) willing to respond to items in a questionnaire, and 4) providing an informed consent. Nursing students/trainees and those who were not have a license to practice nursing in Palestine were not recruited. Participation in this study was voluntary and the study was conducted without financial incentives.

### The questionnaire

The questionnaire used in this study was based on previous studies conducted to assess familiarity of healthcare professionals of issues in ASDs [11, 13, 14, 21]. The questionnaire contained 17 items and was in three sections. The first section contained 8 items to collect the sociodemographic, pedagogic, and practice variables of the practicing nurses who participated in the study like age in years, gender, settings in which nurses were practicing, length of practical experience as a nurse, academic degree in nursing, and place of residence. Nurses were also requested to indicate whether they have taken course(s) on ASDs during their academic nursing degree program or not and whether they have taken a continuing education course/program on ASDs. The second section contained 6 items to collect self-rated familiarity with the signs and symptoms of ASDs, familiarity with the different classes of drugs used to alleviate symptoms of ASDs, familiarity with the specific behavior that could be linked with ASDs that drugs seek to alleviate, the different adverse effects caused by the drugs used to alleviate symptoms of ASDs, familiarity with how to help parents/families/caregivers sort through information to make better informed decisions about their child/patient with ASDs, and familiarity with the allocated resources within the community resources in your region that could be used for referral of a child/patient who is experiencing symptoms that can be linked to ASDs. The nurses had to self-rate the extent to which they were familiar with each item on a Likert scale of 1–5. Scoring 1 indicated complete unfamiliarity and scoring 5 indicated complete familiarity. The third section contained 3 items to collect confidence of the nursing in their abilities to provide counseling for parents/family/caregivers on the drugs prescribed for children/patients with ASDs and their potential adverse effects, whether they disagree/agree that they could benefit from taking a continuing educational/training program on ASDs, and whether they disagree/agree that the nursing school curricula should include courses in the area of ASDs. Again, the nurses had to express their disagreement/agreement on each item on a Likert scale of 1–5. Scoring 1 indicated strong disagreement and scoring 5 indicated strong agreement. Nurses filled the questionnaires in a privacy in their places of work.

### Piloting and reliability testing of the questionnaire

Copies of the questionnaire were distributed to 30 nurses who did not participate in the full study. The nurses were asked to rate the questionnaire for readability, clarity, and compressibility. Some sentences were rephrased to improve readability and comprehensibility. To assess the stability of the scores over a short time period, the test-retest reliability was used. Nurses were requested to complete the questionnaire and after a short period of time (30 min – 2 h), the nurses were requested to complete the questionnaire again. The matching scores in both rounds were compared using Pearson’s correlation. Acceptable stability was ensured by a Pearson’s correlation coefficient (*r*) of > 0.80. The internal consistency (relatedness of the items) was tested using Cronbach’s α statistics. Internally consistent tools should have 0.70 ≤ α ≤ 0.95 [22]. The Pearson’s *r* was 0.93 (95% CI = 0.90–0.95) with a *p* <  0.001 which indicated an excellent stability of scores over a short period of time. The Cronbach’s α was 0.79 which indicated good internal consistency of the items used in the study tool.

### Statistical analysis

Familiarity scores were transformed into percentages as follows: 1 was transformed to 0%, 2 was transformed to 25%, 3 was transformed to 50%, 4 was transformed to 75% and 5 was transformed to 100%. The possible raw familiarity scores could range from 6 to 30 and the possible transformed percentages could range from 0 to 100%. The data were entered into and statistical analyses were conducted using IBM SPSS for Windows, version 21.0 (IBM). Because the sample size was more than 300, absolute skewness and kurtosis values were used to determine whether the data followed normal distribution or not [23, 24]. Absolute skewness values within the range of − 2.0 and + 2.0 and absolute kurtosis within the range of − 7.0 and + 7.0 indicate normally distributed data. In this study, the absolute skewness and absolute kurtosis values were within the range of − 2.0 and + 2.0 and − 7.0 and + 7.0, respectively. The continuous variables like age and length of practical experience were categorized around the mean. Data were expressed as means with their corresponding standard deviation (SD). Differences between familiarity scores among nurses were assessed using Student’s *t*-test. Correlations were investigated using Pearson’s correlation coefficients. To control potentially confounding variables and identify predictors of higher familiarity scores, a multiple linear regression model was used. Variables with a *p*-value of < 0.1 in the Student’s *t*-test and Pearson’s correlations were retaining in the model. The adjusted R^2^ value with a *p*-value of < 0.05 was used to assess the goodness-of-fit of the model. Tolerance values of > 0.1 and variance inflation factors close to 1 indicated absence of multicollinearity issues [25, 26]. In this study, *p*-values of ≤0.05 were considered statistically significant.

### Ethics approval and consent to participate

All procedures performed involving human participants were in accordance with the ethical standards of the institutional and national guidelines. The procedures were also consistent with the ethical principles specified in 1964 Declaration of Helsinki and its later amendments. Studies that are associated with no/minimal risk to the study participants are “Exempt” from review by Institutional Review Boards/Ethics Committees. This study assessed self-rated familiarity with ASDs among nurses and involved no/minimal risk to the study participants. The Institutional Review Board (IRB) of An-Najah National University approved this exemption and the protocol of this study. The study participants provided written informed consent before they took part in the study. Before analysis, data were entered into spreadsheets anonymously.

## Results

### Sociodemographic, pedagogic, and practice variables of the practicing nurses

In this study, the mean age of the nurses was 29.2 years (SD: 7.0) and the mean length of practical experience as a nurse was 7.6 years (SD: 6.9). Of the nurses, more than half (57.4%) were younger than 29 years old and more than 60% were females. The vast majority (about 97%) were nurses practicing in a hospital setting. Of the nurses, 42% were in practice for 7 or more years. The majority (about 84%) had a Bachelor of Science degree in nursing. About 32% of the study participants lived in urban areas. About 55% of the nurses had course(s) on ASDs during their academic nursing degree program and only about 18% had a continuing educational course/program on ASDs. The sociodemographic, pedagogic, and practice variables of the study nurses are shown in Table 1.

**Table 1:** Sociodemographic, pedagogic, and practice variables of the study nurses (*n = 357*)

Variable	n	%
**Age (years)**		
< 29	205	57.4
≥ 29	152	42.6
**Gender**		
Male	141	39.5
Female	216	60.5
**Practice setting**		
Hospital	346	96.9
Others (nursing homes, home care, etc)	11	3.1
**Length of practical experience as a nurse (years)**		
< 7	207	58.0
≥ 7	150	42.0
**Academic degree in nursing**		
Bachelor of Science degree in nursing	298	83.5
Master of Science or other postgraduate degree in nursing	59	16.5
**Place of residence**		
Countryside	242	67.8
Urban area	115	32.2
**Had course(s) on ASDs during the nursing degree program**		
No	162	45.4
Yes	195	54.6
**Had continuing educational course/program on ASDs**		
No	293	82.1
Yes	64	17.9

*ASDs* Autism spectrum disorders, *SD* Standard deviation

### Familiarity of the practicing nurses with signs and symptoms, treatment options, and community resources of ASDs

Nurses expressed the extent of their self-reported familiarity with signs and symptoms, treatment options, and community resources of ASDs. The mean familiarity score was 35.8% (SD: 18.9%). The distribution of the responses of the practicing nurses on each familiarity item is shown in Table 2.

**Table 2:** Familiarity of nurses with symptoms, treatment, and community resources of ASDs

		Not familiar at all		Not familiar		Indecisive		Familiar		Completely familiar	
#	Item	n	%	n	%	n	%	n	%	n	%
1	How would you rate your familiarity with the different signs and symptoms of ASDs?	47	13.2	149	41.7	107	30.0	45	12.6	9	2.5
2	How would you rate your familiarity with the different classes of drugs (e.g., antipsychotic agents, antidepressant agents, central nervous system stimulants) that are used to alleviate symptoms of ASDs?	99	27.7	134	37.5	82	23.0	32	9.0	10	2.8
3	How would you rate your familiarity with the specific behavior that could be linked with ASDs that drugs seek to alleviate (e.g., tendency to self-injury, hyperactivity, and obsessive-compulsive disorder)?	61	17.1	98	27.5	121	33.9	46	12.9	31	8.7
4	How would you rate your familiarity with the different adverse effects caused by the drugs used to alleviate symptoms of ASDs (e.g., extrapyramidal symptoms, irritability, and sedation)?	70	19.6	123	34.5	92	25.8	53	14.8	19	5.3
5	How would you rate your familiarity with how to help parents/families/caregivers sort through information to make better informed decisions about their child/patient with ASDs?	49	13.7	149	41.7	122	34.2	31	8.7	6	1.7
6	How would you rate your familiarity with the allocated resources within the community resources in your region that could be used for referral of a child/patient who is experiencing symptoms that can be linked to ASDs?	103	28.9	124	34.7	85	23.8	28	7.8	17	4.8

*ASDs* Autism spectrum disorders

Nurses were relatively modest in expressing their self-reported familiarity with signs and symptoms, treatment options, and community resources of ASDs. Only 10.4% of the nurses reported familiarity with how to help parents/families/caregivers sort through information to make better informed decisions about their child/patient with ASDs (item # 5) and 12.6% reported familiarity with community resources in their region that can be used for referral of a child/patient who is experiencing symptoms that can be linked to ASDs (item # 6). Again, only 11.8% of the nurses reported familiarity with the different classes of drugs that were used for the treatment of the different symptoms of ASDs (item # 2) and 15.1% reported familiarity with the different sings and symptoms of ASDs (item # 1). Of the nurses, 20.1% reported familiarity with the different adverse effects produced by the drugs used to alleviate symptoms of ASDs (item # 4) and 21.6% reported familiarity with the specific behavior associated with ASDs that drugs seek to alleviate (item # 3).

### Differences in familiarity scores among the nurses

Table 3 shows differences in familiarity scores among the nurses with regard to their sociodemographic, pedagogic, and practice variables. Student’s *t*-test and Pearson’s correlations showed that nurses who were older than 29 years, had 7 or more years of practical experience, had higher degree in nursing, and have had continuing educational course/program on ASDs had significantly higher familiarity scores compared to those who were younger than 29 years, had less than 7 years of practical experience, had a basic degree in nursing, and did not have continuing educational course/program on ASDs (Table 3).

**Table 3:** Differences in familiarity scores among the nurses

			Familiarity score (%)				
Variable	n	%	Mean	SD	***p***-value	Pearson’s r	***p***-value
**Age (years)**							
< 29	205	57.4	33.4	17.1	0.042	0.11	0.042
≥ 29	152	42.6	37.6	20.0			
**Gender**							
Male	141	39.5	37.7	18.1	0.120	−0.08	0.120
Female	216	60.5	34.5	19.4			
**Practice setting**							
Hospital	346	96.9	36.0	18.9	0.212	−0.07	0.212
Others (nursing homes, home care, etc)	11	3.1	28.8	18.3			
**Length of practical experience as a nurse (years)**							
< 7	207	58.0	32.6	17.5	0.006	0.15	0.006
≥ 7	150	42.0	38.2	19.6			
**Academic degree in nursing**							
Bachelor of Science degree in nursing	298	83.5	34.6	18.4	0.007	0.14	0.007
Master of Science or other postgraduate degree in nursing	59	16.5	41.9	20.4			
**Place of residence**							
Countryside	242	67.8	35.3	19.8	0.475	0.04	0.475
Urban area	115	32.2	36.8	17.0			
**Had course(s) on ASDs during nursing degree program**							
No	162	45.4	33.8	20.4	0.064	0.10	0.064
Yes	195	54.6	37.5	17.5			
**Had continuing educational course/program on ASDs**							
No	293	82.1	33.9	18.8	0.000	0.21	< 0.001
Yes	64	17.9	44.5	17.2			

*ASDs* Autism spectrum disorders, *SD* Standard deviation

The multiple linear regression model showed that higher familiarity scores were predicted by having longer practical experience, having a higher academic degree in nursing, and having a continuing educational course/program on ASDs (Table 4).

**Table 4:** Multiple linear regression between sociodemographic and practice variables of the nurses with familiarity scores.

Variable	Unstandardized Coefficients	SE	Standardized Coefficients	t	***p***-value
Age	3.07	3.87	0.08	0.79	0.428
Length of practical experience as a nurse	7.79	3.95	0.20	1.97	0.050
Degree in nursing	8.41	2.64	0.17	3.19	0.002
Had course(s) on autism during nursing degree program	1.99	1.97	0.05	1.01	0.313
Had continuing educational course/program on ASDs	8.66	2.60	0.18	3.33	0.001

*ASDs* Autism spectrum disorders, *SE* Standard error, *t* t-statistics

### Training and confidence of nurses in management of ASDs

Table 5 shows the extend of agreement of the study nurses on 3 statements on their confidence in counseling parents on ASDs and training they need to increase their familiarity with ASDs.

**Table 5:** Training and confidence of nurses in management of ASDs

		Strongly disagree		Disagree		Neutral		Agree		Strongly agree	
#	Item	n	%	n	%	n	%	n	%	n	%
1	I am confident in my abilities to provide counseling for parents/family/caregivers on the drugs prescribed for children/patients with ASDs and their potential adverse effects	100	28.0	127	35.6	98	27.5	17	4.8	15	4.2
2	I could benefit from taking a continuing educational/training program on ASDs	11	3.1	13	3.6	66	18.5	124	34.7	143	40.1
3	The nursing school curricula should include courses in the area of ASDs	10	2.8	8	2.2	48	13.4	78	21.8	213	59.7

Only 9% of the study participants reported that they feel confident in their ability to counsel parents/family/caregivers about the drugs and their adverse effects of prescriptions being used for the treatment of their child/patient with ASDs (Table 5). Interestingly, about 75% of the nurses agreed that they could benefit from taking a continuing educational/training program in the area of ASDs. About 82% of the nurses agreed that the nursing school curriculum should include courses in the area of ASDs.

There was a moderate positive correlation between familiarity scores and confidence in the abilities of the nurses to provide counseling for parents/family/caregivers on the drugs prescribed for children/patients with ASDs and their potential adverse effects (Pearson’s r = 0.45, p-value < 0.001). Similarly, there was a moderate positive correlation between agreement of nurses on benefiting from taking a continuing educational/training program on ASDs and agreement that the nursing school curricula should include courses in the area of ASDs (Pearson’s r = 0.53, *p*-value < 0.001).

## Discussion

The present study investigated self-rated familiarity of practicing nurses of issues related to ASDs. The study also assessed self-rated confidence of nurses in their abilities to provide counseling for parents/family/caregivers on the drugs prescribed for children/patients with ASDs and their potential adverse effects, assessed willingness of nurses to receive education/training on issues in ASDs, and identified the sociodemographic and practice variables that could predict high self-rated familiarity scores.

The comparatively low familiarity scores reported in this study reflected deficits in familiarity of issues in ASDs among practicing nurses in Palestine. Deficits in familiarity of issues in ASDs were previously reported among healthcare professionals including nurses in the Middle East as well as other low- and middle-income countries like Saudi Arabia, Palestine, Egypt, Turkey, and Nigeria [15, 16, 18, 27–29]. In this study, the majority of the practicing nurses reported inadequate familiarity with signs and symptoms, treatment options, and community resources of ASDs. Findings of this study were consistent with those previously reported among practicing pharmacists and medical students in Palestine [18, 19]. In the state of Virginia, a considerable percentage of the surveyed nurses reported inadequate familiarity with the medications used for ASDs [11]. In another study that surveyed nursing faculty reported that nearly 75% of the participants had inadequate familiarity of the drugs used to manage symptoms of ASDs [12]. In the state of Mississippi, practicing pharmacists and pharmacy students also self-reported low familiarity with medications used in the management of ASDs [13, 14]. Apparently nurses in this study self-report higher familiarity with medications used to treat ASDs compared to pharmacists and pharmacy students. This could be explained by the high involvement of nurses in administering medications to patients in all healthcare systems. Compared to other healthcare professionals in Palestine, nurses often provide the largest volume of services including administration of medications to patients, especially in hospitalized patient settings. Therefore, nurses are highly likely to encounter and provide care to patients with ASDs.

In this study, 20.1% of the nurses self-reported adequate familiarity with the different adverse effects caused by drugs used in the management of ASDs symptoms and nearly 21.6% self-reported adequate familiarity with the specific behavior that could be linked to ASDs that the drugs see to alleviate. Currently, aripiprazole and risperidone are approved to alleviate the behavioral symptoms that could be linked to ASDs. It is noteworthy mentioning that behavioral problems in ASDs are often managed using potent psychotropic medications [30]. Buspirone was shown to be effective as adjunct therapy for the restrictive and repetitive behavior, especially when it is used in addition to behavioral interventions in young children with ASDs [31]. In a study conducted in Northern New England, use of psychotropic medications was 9-fold higher in children with ASDs compared to general population [32]. Medications used in children with ASDs can cause serious side effects. For example, psychotropics are associated with tardive dyskinesia, weight gain and sedation [33]. In many cases, nurses are the first to witness and report a side effect. Therefore, nurses should be familiarity with and knowledgeable of the side effects caused by medications used to treat children with ASDs in order to help manage these side effects [34]. Findings of this study showed that practicing nurses in Palestine self-report higher familiarity with medications different adverse effects caused by drugs used in the management of ASDs symptoms and the specific behavior that could be linked to ASDs that the drugs see to alleviate compared to Palestinian medical students and pharmacists [18, 19]. In Virginia, about 46% of the nurses surveyed reported familiarity with the adverse effects caused by the drugs used to alleviate symptoms of ASDs and, similarly, 46% reported familiarity with the specific behavior that could be linked to ASDs [11]. Taken together, practicing nurses might have encountered and administered medications to more patients with ASDs compared to medical students and pharmacists [12].

Given the increasing prevalence of ASDs in different populations, it is highly likely that medications use will also increase. This will present a challenged for healthcare professionals including nurses. As nurses often interact with parents or caregivers of patients with ASDs, it is highly expected that they need to counsel parents on ASDs and the medication used to alleviate the behavioral symptoms associated with ASDs. A recent qualitative study in Egypt reported high information-seeking behavior among parents of children with ASDs [10]. This seems to be challenging in the current situations as the majority of the nurses in this study self-reported inadequate familiarity with the different signs and symptoms of ASDs. This also could be concerning as the majority of the practicing nurses self-reported inadequate familiarity with the resources allocated within the community in their regions that could be used for referral of children/patients with ASDs who are experiencing symptoms that could be linked to ASDs and a comparable percentage of the nurses self-reported some familiarity on how help parents/families/caregivers sort through information to make better informed decisions about their child/patient with ASDs. In previous studies, Palestinian medical students and practicing pharmacists in Palestine also reported inadequate familiarity with the resources that could be used for referral of children/patients with ASDs and how help parents/families/caregivers sort through information make better informed decisions about their child/patient with ASDs [18, 19]. Findings of this study were not surprising as 91% of the practicing nurses were not confident in their abilities to provide counseling to parents/families/caregivers about the drugs prescribed to children/patients with ASDs and their potential adverse. Lack of familiarity with community resources and how to help families of patients with ASDs sort through information was also reported among nurses in other low- and middle-income countries as well as high income countries like Nigeria, Turkey, and the US [11, 15, 29]. About 81% of the nurses surveyed in Virginia were minimally familiar with how to help parents/families/caregivers sort through information and only about 31% were familiar with resources allocated for ASDs within the communities in their region [11]. Many nurses in the study of Gardner and colleagues were not prepared to counsel family members of the patients with ASDs [12]. As ASDs are still puzzling disorders, parents/families/caregivers of children/patients with ASDs would rely on healthcare professionals including nurses to learn more about ASDs. Parents/families/caregivers would expect the practicing nurses to help them sort through information to make better informed decisions about their children/patients with ASDs and to refer them to resources allocated for patients with ASDs within the communities in their regions. Nurses who are not familiar with these resources would fail to do so. Therefore, nurses should assume responsibility and increase their familiarity with and knowledge of issues in ASDs.

In this study, the length of practical experience, higher academic degree in nursing, and having a continuing education program on ASDs were significantly associated with higher familiarity scores. These findings were not surprising as nurses with longer experience might have encountered more patients with ASDs. On the other hand, nurses who have had higher academic degree in nursing might have had received more didactics/education on ASDs. Additionally, nurses who have had a specific continuing education program on ASDs might have become more aware and familiar with ASDs [16]. In many cases, continuing education programs increased knowledge in certain domains [16, 35, 36]. Griscti and Jacono reviewed the effectiveness of continuing education programs in nursing, in their review, continuing education programs were demonstrated to increase familiarity as well as initiatives to keep knowledge and skills of nurses up to date [36, 37]. Many studies have shown that when comparing self-perceived competences of nurses who attended a continuing education program and those who did not attend, there were statistically significant differences in favor of those who attended a continuing education program [36–38].

Findings of this study highlighted considerable familiarity deficits among practicing nurses with regard to ASDs. The sociodemographic, pedagogic, and practice variables that were associated with high and low familiarity were determined. Results of this study might be important for decision and policy makers who might wish to plan/design interventions to promote familiarity of nurses with issues in ASDs. It has been argued that better informed nurses might be able to provide higher quality services and care to patients with ASDs and/or their families/caregivers.

### Strengths and limitations

Findings of this study might be interpreted after considering a number of strength points and limitations. First, this is the first assessment of familiarity of ASDs among practicing nurses in Palestine. Second, the questionnaire used in this study was previously used to assess familiarity of ASDs among practicing pharmacists and pharmacy students in Palestine and Mississippi [13, 14, 18]. Items in the questionnaire were adapted from previous questionnaires used to assess familiarity of ASDs among school nurses and speech-language pathologists [11, 21]. In today’s healthcare practice, people with ASDs receive services from different providers including nurses, general practitioners, psychotherapists, psychiatrists, behavioral therapists, occupational therapists, speech-language pathologists, and social workers [16]. The items used in previous questionnaires were adapted to the services provided by the healthcare providers to patients with ASDs [11, 13, 14, 16, 18, 21]. In this study, the items used in the questionnaire were adapted to the roles of nurses in caring for patients with ASDs. Third, the necessary diagnostics were used to reassess the study tool for reliability and internal consistency. Fourth, the number of nurses who participated in this study was larger than the sample size that was needed for this study. Fifth, the practicing nurses who participated in this study were recruited from different healthcare centers in the West Bank of Palestine. Finally, appropriate statistical methods were used to determine what predicted higher familiarity scores among the practicing nurses.

This study has a number of limitations. First, the construct of familiarity equally weighed the 6 items used to measure this construct. Of those 6 items, 3 (50%) were related to familiarity with classes of drugs used to alleviate symptoms of ASDs, their side effects, and the specific behaviors that the drugs seek to alleviate. This arbitrary weighing could have affected the construct of familiarity measured in this study. The other items measured familiarity with signs and symptoms of ASDs, how to help parents/families/caregivers sort through information, and familiarity with the allocated resources that could be used for referral of a child/patient with ASDs. It is noteworthy mentioning that the use of prescription and nonprescription drugs is significantly higher among patients with ASDs compared to their age-, sex-, and race-matched cohorts without ASDs [39]. In clinical practice, nurses are responsible for preparing administering drugs to patients including those with ASDs. Therefore, nurses should be knowledgeable with the drugs and the specific behaviors that the drugs seek to alleviate. Moreover, nurses should be able to screen for and recognize side effects of the drugs [40]. Therefore, more arbitrary emphasis was placed on familiarity of nurses with the drugs used to manage patients with ASDs in this study. Second, familiarity in this study was self-reported. It could have been better if knowledge was investigated as additional domain in this study. Third, the study was conducted in a cross-sectional observational design. No intervention was conducted to increase familiarity of the nurses with regard to ASDs. Fourth, a nonprobability sampling technique was used to recruit the nurses in this study. Nonprobability sampling techniques are inherently biased when compared to probability sampling techniques. Over- or under-estimation of familiarity and knowledge could not be ruled out as a result of using this nonprobability sampling technique. Fifth, although the nurses were asked to specify the settings in which they practiced, nurses were not asked to specify which services/wards they served in. Collection of such information should have allowed comparing familiarity scores of nurses who practiced in psychiatry and pediatric services/wards to those of nurses who served in other services/wards. Finally, the sociodemographic, pedagogic, and practice variables of the nurses who declined to participate in this study were not collected. However, the response rate obtained in this study was relatively high. Additionally, the sample included nurses of both genders, different practice settings, geographical locations, age groups, academic degrees, and length of practicing experience. This should have minimized the possibility of lack of representation of certain subgroups of nurses in the Palestinian nursing practice.

## Conclusion

In conclusion, findings of this study highlighted inadequate familiarity with issues of ASDs among practicing nurses. Nurses expressed low confidence in their ability to counsel parents of children with ASDs on how to sort information and showed little familiarity with community resources devoted to ASDs in their regions. Higher familiarity was predicted by the length of practical experience, higher academic degree in nursing, and having a continuing educational course/program on ASDs. Specifically designed pedagogic interventions might be helpful in increasing familiarity of practicing nurses on ASDs. More investigations are still needed to evaluate if these interventions can improve familiarity and services provided to patients with ASDs.

## Supplementary Information

**Additional file 1.**

## Data Availability

All the data relevant to this work are included within the manuscript or provided as supplementary materials. In case the datasets used and/or analyzed during the current study are needed, they are available from the corresponding author on reasonable request.
