# Supplementary material for: Self-rated familiarity with autism spectrum disorders among practicing nurses: a cross-sectional study in the palestinian nursing practice
Source: BMC Nurs. 2021 Dec 3;20:241. doi: 10.1186/s12912-021-00764-3 (PMC8642987; doi:10.1186/s12912-021-00764-3)
Supplement: Supplementary file 1 — Additional file 1. [file 12912_2021_764_MOESM1_ESM.docx]

**Additional file for the manuscript:**

**Self-rated familiarity with autism spectrum disorders among practicing nurses: a cross-sectional study in the Palestinian nursing practice**

Ramzi Shawahna^1,2,3*^

^1^Department of Physiology, Pharmacology and Toxicology, Faculty of Medicine and Health Sciences, An-Najah National University, Nablus, Palestine

^2^An-Najah BioSciences Unit, Centre for Poisons Control, Chemical and Biological Analyses, An-Najah National University, Nablus, Palestine

**^*^Corresponding author:**

Ramzi Shawahna, PhD, Department of Physiology, Pharmacology and Toxicology, Faculty of Medicine & Health Sciences, New Campus, Building: 19, Office: 1340, An-Najah National University, P.O. Box 7, Nablus, Palestine

Phone: + (970) 923 45113 ext 2772

Phone: + (970) 92349739

Email: [ramzi_shawahna@hotmail.com](mailto:ramzi_shawahna@hotmail.com)

**Supplementary Table S1:** Adherence to STROBE Statement for reporting cross-sectional studies [[1](#_ENREF_1)]

| **Section** | **Item #** | **Recommendation** | **Section** |
| --- | --- | --- | --- |
| **Title and abstract** | 1 | (*a*) Indicate the study’s design with a commonly used term in the title or the abstract | The title indicates that the study was a cross-sectional study |
|  |  | (*b*) Provide in the abstract an informative and balanced summary of what was done and what was found | The abstract is an informative and balanced summary of what was done and what was found. The abstract adheres to the style of the journal. |
| **Introduction** | | | |
| Background/rationale | 2 | Explain the scientific background and rationale for the investigation being reported | Provided in the Background section. |
| Objectives | 3 | State specific objectives, including any pre-specified hypotheses | Provided in the Background. Please see the last paragraph. |
| **Methods** | | | |
| Study design | 4 | Present key elements of study design early in the paper | Provided in the Methods section, under Design of the study. |
| Setting | 5 | Describe the setting, locations, and relevant dates, including periods of recruitment, exposure, follow-up, and data collection | Provided in the Methods section, under Study nurses and sampling |
| Participants | 6 | (*a*) Give the eligibility criteria, and the sources and methods of selection of participants | Provided in the Methods section, under Study nurses and sampling |
| Variables | 7 | Clearly define all outcomes, exposures, predictors, potential confounders, and effect modifiers. Give diagnostic criteria, if applicable | Provided in the Methods section, under the Questionnaire |
| Data sources/ measurement | 8* | For each variable of interest, give sources of data and details of methods of assessment (measurement). Describe comparability of assessment methods if there is more than one group | Provided in the Methods section, under the Questionnaire |
| Bias | 9 | Describe any efforts to address potential sources of bias | Provided in the Methods section, under Sample size estimation and Piloting and reliability testing of the questionnaire. |
| Study size | 10 | Explain how the study size was arrived at | Provided in the Methods section, under Study nurses and sampling |
| Quantitative variables | 11 | Explain how quantitative variables were handled in the analyses. If applicable, describe which groupings were chosen and why | Provided in the Methods section, under Statistical analysis. |
| Statistical methods | 12 | (*a*) Describe all statistical methods, including those used to control for confounding | Provided in the Methods section, under Statistical analysis. |
|  |  | (*b*) Describe any methods used to examine subgroups and interactions | Provided in the Methods section, under Statistical analysis. |
|  |  | (*c*) Explain how missing data were addressed | Not applicable |
|  |  | (*d*) If applicable, describe analytical methods taking account of sampling strategy | Not applicable |
|  |  | (*e*) Describe any sensitivity analyses | Not applicable |
| **Results** | | | |
| Participants | 13* | (a) Report numbers of individuals at each stage of study—eg numbers potentially eligible, examined for eligibility, confirmed eligible, included in the study, completing follow-up, and analysed | Provided in the Results section. subsection: Sociodemographic, pedagogic, and practice variables of the practicing nurses and Table 1. |
|  |  | (b) Give reasons for non-participation at each stage | Not applicable. |
|  |  | (c) Consider use of a flow diagram | Not applicable. |
| Descriptive data | 14* | (a) Give characteristics of study participants (eg demographic, clinical, social) and information on exposures and potential confounders | Provided in the Results section. subsection: Sociodemographic, pedagogic, and practice variables of the practicing nurses and Table 1. |
|  |  | (b) Indicate number of participants with missing data for each variable of interest | Not applicable. |
| Outcome data | 15* | Report numbers of outcome events or summary measures | Table 1. |
| Main results | 16 | (*a*) Give unadjusted estimates and, if applicable, confounder-adjusted estimates and their precision (eg, 95% confidence interval). Make clear which confounders were adjusted for and why they were included | Tables in the results section |
|  |  | (*b*) Report category boundaries when continuous variables were categorized | Tables in the results section |
|  |  | (*c*) If relevant, consider translating estimates of relative risk into absolute risk for a meaningful time period | Not applicable. |
| Other analyses | 17 | Report other analyses done—eg analyses of subgroups and interactions, and sensitivity analyses | Results section and Tables in the results section |
| **Discussion** | | | |
| Key results | 18 | Summarise key results with reference to study objectives | Provided in Discussion section. Please see the 1^st^ paragraph |
| Limitations | 19 | Discuss limitations of the study, taking into account sources of potential bias or imprecision. Discuss both direction and magnitude of any potential bias | Provided in Discussion, under Strengths and Limitations |
| Interpretation | 20 | Give a cautious overall interpretation of results considering objectives, limitations, multiplicity of analyses, results from similar studies, and other relevant evidence | Provided in the Discussion section. |
| Generalisability | 21 | Discuss the generalisability (external validity) of the study results | Provided in Discussion, under Strengths and Limitations |
| **Other information** | | | |
| Funding | 22 | Give the source of funding and the role of the funders for the present study and, if applicable, for the original study on which the present article is based | Provided in the Declarations |

**Reference**

1. Vandenbroucke JP, von Elm E, Altman DG, Gotzsche PC, Mulrow CD, Pocock SJ, Poole C, Schlesselman JJ, Egger M: **Strengthening the Reporting of Observational Studies in Epidemiology (STROBE): explanation and elaboration**. *PLoS medicine* 2007, **4**(10):e297.
